# Volatile and Nonvolatile Constituents and Antioxidant Capacity of Oleoresins in Three Taiwan Citrus Varieties as Determined by Supercritical Fluid Extraction

**DOI:** 10.3390/molecules21121735

**Published:** 2016-12-17

**Authors:** Min-Hung Chen, Tzou-Chi Huang

**Affiliations:** Department of Food Science, National Pingtung University of Science & Technology, Pingtung 90090, Taiwan; e933623366@yahoo.com.tw

**Keywords:** oleoresin, supercritical fluid extraction, volatile compounds, antioxidant capacity

## Abstract

As local varieties of citrus fruit in Taiwan, Ponkan (*Citrus reticulata* Blanco), Tankan (*C. tankan* Hayata), and Murcott (*C. reticulate* × *C. sinensis*) face substantial competition on the market. In this study, we used carbon dioxide supercritical technology to extract oleoresin from the peels of the three citrus varieties, adding alcohol as a solvent assistant to enhance the extraction rate. The supercritical fluid extraction was fractionated with lower terpene compounds in order to improve the oxygenated amounts of the volatile resins. The contents of oleoresin from the three varieties of citrus peels were then analyzed with GC/MS in order to identify 33 volatile compounds. In addition, the analysis results indicated that the non-volatile oleoresin extracted from the samples contains polymethoxyflavones (86.2~259.5 mg/g), limonoids (111.7~406.2 mg/g), and phytosterols (686.1~1316.4 μg/g). The DPPH (1,1-Diphenyl-2-picrylhydrazyl), ABTS [2,2′-azinobis-(3-ethylbenzothiazoline-6-sulfonic acid)] scavenging and inhibition of lipid oxidation, which test the oleoresin from the three kinds of citrus, exhibited significant antioxidant capacity. The component polymethoxyflavones contributed the greatest share of the overall antioxidant capacity, while the limonoid and phytosterol components effectively coordinated with its effects.

## 1. Introduction

Collectively, citrus trees comprise the most abundant crop of fruit trees in the whole world, providing fruits which have a high commercial value on both the fresh market and the food processing industry [1]. At present, the citrus varieties produced in Taiwan are the Ponkon (*Citrus reticulata* Blanco), Tankan (*C. tankan* Hayata) and Murcott (*C. reticulate* × *C. sinensis*) varieties, with 108,058 tons of Ponkon, 52,023 tons of Tankan, and 24,751 tons of Murcott having been produced in one recent year [2]. The harvest months for Ponkon, Tankan, and Murcott are October–December, February–March, and January–March, respectively. The peels and pulps of processed citrus product waste contain high levels of active biological components, including carotenoids, vitamin E, flavonoids, limonoids, phenols, polysaccharides, lignin, cellulose, pectin, and essential oils [3,4,5].

Essential oils and oleoresin are found in citrus peels, the bulk of which are mainly obtained from the byproducts of the juice manufacturing industry, and are extracted for use in beverages and cosmetics with approximately 56,200 tons being used annually [6,7]. Extracted in different manners with different solvents, essential oils are the volatile aromatic plants prepared by steam distillation; oleoresins are extracts of spices obtained by using organic solvents, after the solvent is removed from the extract. Oleoresin itself is composed of non-volatile resins and volatile essential oil. According to several studies, a variety of the non-volatile oleoresin compounds, such as piperine from black pepper oleoresin, gingerol from ginger oleoresin, and lycopene from tomato oleoresin, can strengthen sensory stimulation [8,9,10]. Supercritical fluid extraction (SFE) is a relatively new technology that has been widely used in recent years in producing perfumes and spices, as well as in the extraction of active ingredients, particularly plant extracts. SFE is regarded as a green technology due to the fact that it is low cost, high purity, nontoxic, and solvent-free [11,12,13,14,15]. The use of SFE to extract oleoresin can produce oleoresin with an enhanced flavor and more intense aroma, and its use also yields oleoresin with improved storage stability because it prevents the degradation of thermally unstable components during the extraction process [13,14,15].

The specific properties of a given sample of citrus essential oil and oleoresin will affect its quality in terms of its utility for industrial applications, and these properties depend in turn, at least in part, on the origin of the raw material used (including the different fruit varieties of which it consists) and the different extraction processes used. Volatile essential oils of plants can be divided into three categories: monoterpenes, sesquiterpenes, and oxygenated terpenes. Essential oils extracted via the cold-pressing method contain at least 200 compounds and some natural pigments, such as chlorophyll and carotenoids [16,17,18,19,20]. The flavor characteristics of cold-pressed citrus essential oils are associated with the contents of the oil, consisting of terpene compounds and oxygen-containing derivatives. Among those contents, volatile components account for about 96%~98% of the total volume, whereas non-volatile compounds, such as long-chain hydrocarbons, fatty acids, sterols, carotenoids, and oxygenated heterocyclic compounds, account for about 1%. The oxygenated heterocyclic compounds, including coumarins, furanocoumarins, and flavonoids, are associated with essential oil safety and efficacy, as well as tests to determine essential oil authenticity [17,18,19]. The non-volatile compounds in citrus oils account for an extremely low portion of their total content, but these non-volatile compounds are nonetheless highly important, with key compounds strongly determining the quality of a given oil sample in terms of sensory evaluations [20].

The purpose of the present study was to evaluate the quality and characteristics of oleoresin extracted via SFE from the main varieties of citrus produced in Taiwan, namely the Ponkan, Tankan and Murcott varieties. More specifically, this study analyzed the oleoresin from these varieties in order to determine their composition of volatile and non-volatile compounds and its antioxidant capacity.

## 2. Results and Discussion

### 2.1. Analysis of Volatile Essential Oils

Many studies have pointed out that the use of ethanol as a co-solvent can enhance the efficiency and range of SFE, effectively increasing the hydrophilic compound selectivity of citrus peel. Because ethanol contains a hydroxyl group that can form hydrogen bonds with other groups, it attracts the polar molecules of citrus peel when used in SFE [13,14,15]. In this study, 10% ethanol was used as a co-solvent in SFE, and the results showed that the respective oleoresin extraction rates for the Ponkon, Tankan, and Murcott citrus varieties were 1.1%, 2%, and 0.8% (*w*/*w*).

Citrus oleoresin extracted via SFE is composed of volatile compounds, a fact which can be applied in identifying specific citrus species when utilizing gas chromatography/mass spectrometry (GC/MS) for composition identification. The results of the present study indicated that, across the different samples tested, 33 volatile compounds were identified, 23 of which were present in the Ponkan samples, 27 of which were present in the Tankan samples, and 28 of which were present in the Murcott samples. Divided by functional groups, these compounds consisted of 11 kinds of monoterpenes, 7 kinds of sesquiterpenes, 9 kinds of aldehydes, 3 kinds of alcohols, 2 kinds of esters, and 1 kind of ketone (Table 1).

The terpene compounds of citrus oleoresin do not contribute to its flavor and fragrance. However, these terpene compounds are the main components of oleoresin, accounting for 95.11% of Ponkan oleoresin, 96.75% of Tankan oleoresin, and 94.92% of Murcott oleoresin. The main monoterpenes in the oleoresin samples from the three citrus varieties were limonene, α-pinene, and β-myrcene. The reaction mechanisms of all monoterpene synthases start with the ionization of the geranyl diphosphate (GPP) substrate. The resulting carbocation can undergo a range of cyclizations, hydride shifts, and rearrangements before the reaction is terminated by deprotonation or water capture [21,22,23]. Myrcene is generated by losing the geranyl cation of protons, limonene is produced by losing an α-terpinyl cation, and when the double bond of the α-terpinyl cation is attacked by an electron, an α-pinene is produced [21]. For the oleoresin from all three citrus varieties, limonene was the most abundant terpene compound, accounting for 72.72%~86.13% of the total fraction (Table 1), whereas it has previously been found to account for 90.5%~94.6% of the total fraction of oleoresin extracted from oranges by cold pressing [24,25,26]. Due to the terpenes themselves being extremely unstable in light and heat, they are prone to deterioration when placed in storage, causing a loss of flavor and overall quality. Deterpenation is thus an important aspect of citrus oil processing, with the fractionation of lower terpene compounds allowing producers to limit product deterioration [13,26].

The oxygenated compounds are generally preferable due to their characteristic flavors [13,16,26], which are effectively used as indicators of higher quality. For the Ponkon, Tankan, and Murcott oleoresin samples in this study, oxygenated compounds accounted for 4.89%, 3.25%, and 5.08% of the total fractions. The Ponkon oleoresin had 10 oxygenated compounds, including nonanal, decanal, perillaldehyde, undecanal, citronellal, neral, β-sinensal, linalool, α-terpineol, and carvone. The Tankan oleoresin had 11 oxygenated compounds including nonanal, decanal, 2-decenal, perillaldehyde, β-sinensal, α-sinensal, linalool, α-terpineol, geranyl acetate, citronellyl acetate, and neryl acetate. The Murcott oleoresin had 12 oxygenated compounds including nonanal, decanal, perillaldehyde, undecanal, citronellal, neral, α-sinensal, linalool, α-terpineol, citronellyl acetate, neryl acetate, and carvone (Table 1). Because of a preference for such oxygenated compounds, the content of oxygenated compounds has become an important parameter in determining the price of citrus oils [21].

The compositional variations of terpene aldehydes in citrus oils can be used to distinguish the oils [25]. In this study, the aldehyde components of the samples were further categorized into 3 kinds of aliphatic aldehydes, 3 kinds of monoterpene aldehydes and 3 kinds of sesquiterpene aldehydes (Table 1). Aliphatic aldehydes convey a sweet waxy aroma and citrus peel–like odor due to the active compounds of citrus, including nonanal, decanal, and undecanal. Terpene alcohols reveal a fresh top-note and floral aroma [27]. The fractions of the alcohol compounds in the citrus oil samples from Ponkan, Tankan, and Murcott were 0.81%, 1.40%, and 1.05% (Table 1). Linalool has a light citrus note and woody floral aroma and has usually been reported as the most prominent alcohol of citrus oils. Linalool is difficult to separate from terpenes, and due to this fact it is considered the active component via deterpenation [13,16]. The formation of linalool, myrcene, and (*E*)-β-ocimene might proceed either via the geranyl cation or via the linalyl cation. The numbering of carbon atoms of intermediates and products refers to that for GPP [21]. Some oxygenated terpene compounds, such as carvone and α-terpineol oxidized from limonene, induce negative flavor influences [28]. Moreover, some past research studies have indicated that limonene, α-pinene, myrcene, γ-terpinene, and linalool have the highest flavor dilution values. Among them, linalool, α-terpineol, and decanal are odor-active compounds [29].

### 2.2. Analysis of Non-Volatile Resins

**Figure 1:**
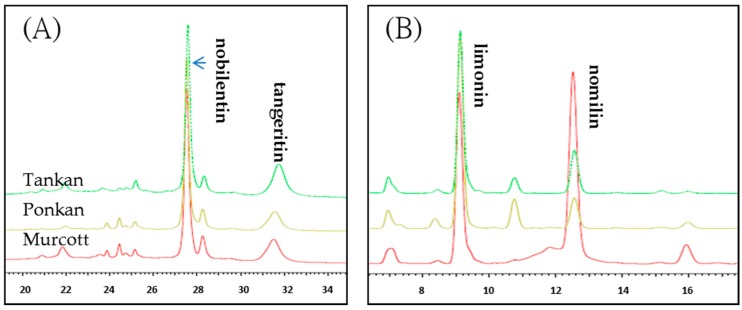
Analysis of flavonoid (**A**) and limonoid (**B**) by HPLC.

Some flavonoid derivatives in citrus are specific. In this study, the components of citrus oleoresin extracted with SFE were analyzed by high performance liquid chromatography (HPLC), but phenolic acid and flavonoid aglycone were not detected. SFE cannot extract polar compounds; thus, polymethoxyflavone (PMF) could be detected in this study because of its lipophilic properties (Figure 1). In contrast, some related studies have reported that the PMF in the citrus oil of fortified commercial citrus juice cannot be detected [30,31].

The literature reported finding rich flavonoids in the Taiwan citrus peel, and hesperidin was the most prevalent of these, with Ponkan and Tonkan peels containing 29.5 and 23.4 mg/g dry powder, respectively [32]. In this study, the identified flavonoids were tangeritin and nobilentin. Tangeritin and nobilentin both have many methoxy groups. The bioactivities of PMF, such as anti-inflammation, anticancer, anti-atherosclerosis, anti-virus, and anti-oxidative activities, are better than those of citrus flavonoids [33,34,35]. The tangeritin levels in Ponkan oleoresin, Tankan oleoresin, and Murcott oleoresin found in this study were 64.12, 54.19, and 24.05 mg/g. The nobilentin levels in Ponkan oleoresin, Tankan oleoresin, and Murcott oleoresin found in this study were 195.45, 99.31, and 62.23 mg/g.

Citrus fruits contain abundant levels of phytosterols and limonoids, which have been identified to have many physiological functions. Limonoids have antibacterial, antipyretic, antimalarial, anticancer, and antiviral properties, in addition to begin able to promote glutathione S-transferase activity in liver and small intestinal mucosa [36]. Limonoids can be lactonized by the action of enzymes, giving them a slightly bitter taste [36]. The results of this study of citrus oleoresin extracted by SFE found that the samples were rich in limonoids, with the limonin levels in the Ponkan, Tankan, and Murcott being 71.17, 316.02, and 117.83 mg/g and the nomilin contents being 41.53, 90.24, and 130.47 mg/g, respectively. The Ponkan contained the highest amount of limonoids (Table 2).

The pertinent literature has demonstrated that phytosterols have anti-inflammatory, anticancer, and antibacterial properties. Moreover, phytosterols can affect the structure of cell membranes and host organizations, including via the message pathway involved in the regulation of tumor growth and differentiation, in order to inhibit the growth of cancer cells [37]. Related studies have shown that citrus peel is a good source of phytosterols (23.4~32.6 mg/100 g dry powder) [38]. In this study, the total phytosterol level was the highest in the Murcott oleoresin (1216.42 μg/g), with the levels for the Ponkan and Tankan oleoresin being 985.54 and 876.88 μg/g, respectively (Table 2). The analysis results identified four kinds of phytosterols in the citrus oleoresin extracted via SFE. The Ponkan oleoresin contained campesterol (304.77 μg/g) and stigmasterol (680.67 μg/g); the Tankan oleoresin contained campesterol (211.61 μg/g), stigmasterol (131.82 μg/g), lanosterol (189.33 μg/g), and fucosterol (153.34 μg/g); and the Murcott oleoresin contained campesterol (455.54 μg/g), stigmasterol (374.17 μg/g), and fucosterol (486.71 μg/g).

### 2.3. Antioxidant Properties

**Figure 2:**
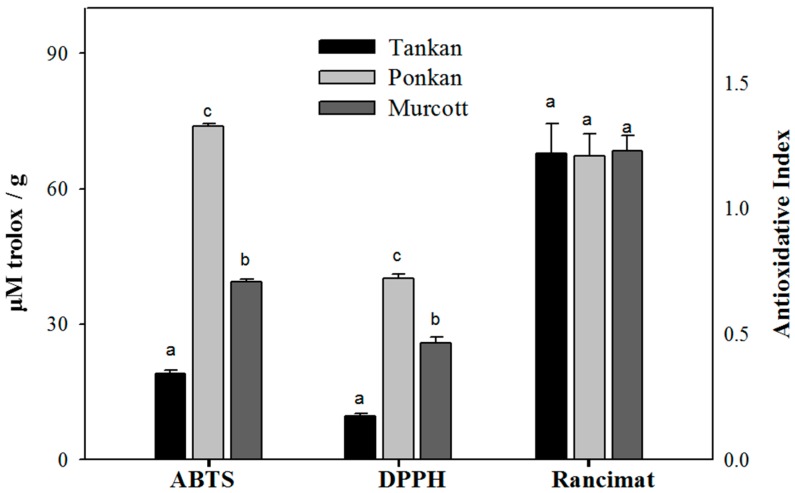
ABTS and DPPH scavenging abilities and antioxidative indexes of citrus oleoresin. ^a^ Data presented are in mean ± SD (*n* = 3), with the different letters indicating significant differences (*p* < 0.05).

The results of this study further showed that the 1,1-diphenyl-2-picrylhydrazyl (DPPH) clearing capacity of the citrus oleoresin extracted by SFE was 9.69~40.15 μM Trolox/g, while the 2,2′-Azinobis(3-ethylbenzothiazolin-6-sulfonic Acid) (ABTS) clearing capacity was 19.07~73.80 μM Trolox/g (Figure 2). The Pokan oleoresin had the best antioxidant capacity among the three varieties. In addition, in terms of the essential oils extracted with steam distillation, the scavenging DPPH capacity of the Pokan oils was 0.12~0.19 μM Trolox/mL, while the ABTS clearing capacity was 0.31~0.37 μM Trolox/mL (data not shown). Essential oils have poorer antioxidant capacity than citrus oleoresin because the terpenoid compounds in essential oils accelerate oxidant reactions.

**Figure 3:**
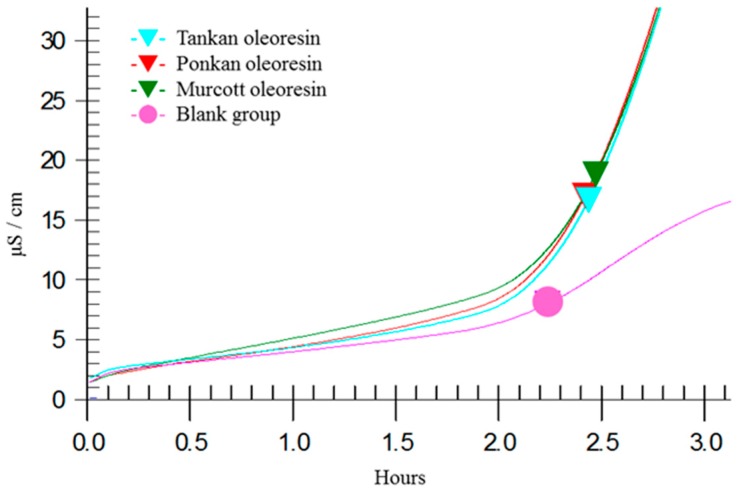
Oxidation curves obtained for the oleoresin samples extracted from the three citrus varieties as determined by Rancimat.

The independent or complex combinations of antioxidants from oil are able to slow down the evolution of peroxide compounds effectively under Rancimat testing conditions [39]. In this study, citrus oleoresin extracted via SFE was dissolved in sunflower oil at 1 mg/mL, and the results showed that the sunflower oil group prolonged the induction time for 2.2 h during the evolution of peroxide compounds, such that citrus oleoresin extracted via SFE was able to extend the induction time to 2.42~2.47 h (Figure 3), with the obtained antioxidative indexes for the Ponkan, Tankan, and Murcott extracts being 1.21, 1.21, and 1.23, respectively (Figure 2). However, the antioxidative indexes of the essential oils extracted via steam distillation were <0.8 (data not shown). The experimental results indicated that all of the citrus-extracted oleoresins exerted fat-oxidative inhibition, although there were no differences in the three citrus varieties.

Flavonoids are powerful antioxidants against free radicals because they act as radical scavengers [34,35]. This activity is attributed to their hydrogen-donating ability. Three structural groups are important for the evaluation of their antioxidant capacity: (I) the ortho-dihydroxy structure in the B-ring, which confers greater stability to radicals, possibly through hydrogen bonding, and which participates in electron dislocation; (II) the 2,3-double bond, which, in conjugation with a 4-oxo function, is responsible for electron dislocation from the C-ring; and (III) the 3-hydroxyl group in the C-ring [34].

According to the structures of tangeritin and nobilentin, in comparison to flavonoids, PMF has fewer hydroxyl groups, revealing that the 2,3-double bond conjugated with a 4-oxo function, which is responsible for electron dislocation from the C-ring, could be responsible for free radical clearness. However, the DPPH scavenging effects do appear to be under the influence of the structure [40]. The results of this study revealed that citrus extract fared better in terms of scavenging for ABTS.

The results also indicated the citrus oleoresins were rich in limonoids (11.2%~40.6%) and PMF (8.6%~25.9%). Limonoids are highly oxygenated triterpenoids with fewer hydroxyl groups than flavonoids, such that their free radical scavenging ability is poor [41]. The results of this study revealed that antioxidant ability is positively associated with the PMF content in citrus oleoresin. Other studies have shown that the protection of antioxidants can prevent the degradation of phytosterol; however, while antioxidants are important, the interactions between the different compounds and their compositions are also important [42]. The citrus peel compounds extracted by supercritical fluid technology in this study were highly pure in terms of their limonin, PMF, and phytosterol contents, the active ingredients of which have been shown to have significant physiological effects, in addition to synergistic effects between themselves.

## 3. Materials and Methods

### 3.1. Plant Materials and Sample Preparation

Three varieties of citrus fruits, namely Ponkan (*C. reticulate* Blanco), Tankan (*C. tankan* Hayata), Murcott (*C. reticulate* × *C. sinensis*), were harvested from trees at local farms in January 2016. The citrus fruits were separated into edible and inedible portions (peel), and the peel was dried under a warm stream (below 50 °C) and milled in a grinder (IKA-Werke GmbH & Co. KG, Staufen, Germany) to produce powder which was passed through a 20-mesh sieve. The particle size was 0.8 mm, and the powder was finally stored at −30 °C before use.

### 3.2. Samples and Reagents

ABTS, DPPH, Trolox (6-hydroxy-2,5,7,8-tetramethylchroman-2-carboxylic acid), tangeritin, and nobilentin were purchased from Sigma Chemical Co. (St. Louis, MO, USA). All other analytical grade chemicals and ethanol were purchased from Echo Chemical Co., Ltd. (Miaoli, Taiwan).

### 3.3. SFE Using Carbon Dioxide and Ethanol

A supercritical fluid extractor Spe-ed SFE-2 (Applied Separations, Inc., Allentown, PA, USA), which operates with two pumps, including a master pump fitted with a cooling jacket on the pump head and a second pump (Knauer pump, model K-501, Berlin, Germany) for the addition of an organic modifier, was used. For extraction via SFE, 50 g dried citrus peel powder was placed in an extraction vessel. Aqueous ethanol (95%) was chosen as a modifier in this study. The extraction was started when the desired pressure of 20 Mpa and the specified temperature of 50 °C were reached for 1 h. The operating pressure was provided by an air compressor. The extracted analyte was collected in a glass vial with a rubber plug at the top. The CO_2_ flow rate was kept at approximately 6 mL/min by adjusting the outlet valve manually.

### 3.4. GC/MS Spectrometry Analysis

The volatile compounds were identified using an Agilent 6890 GC equipped with a 60 m × 0.25 mm i.d. DB-1 fused-silica capillary column (Agilent, Palo Alto, CA, USA) with a film thickness of 0.25 μm coupled to an Agilent model 5973 N MSD mass spectrometer The injector temperature was maintained at 250 °C. The split ratio was set at 50:1. The initial temperature was set at 40 °C for 10 min, then programmed at 2 °C/min up to 240 °C, and held at this temperature for 20 min. The carrier gas (helium) flow rate was 1 mL/min. The electron energy was 70 eV at 230 °C. The constituents were identified by matching their spectra with those recorded in a mass spectral library (Wiley 7n). In addition, the constituents were confirmed by comparing the Kovats indices or GC retention time data with those of authentic standards or those in the published literature. The linear RIs were calculated from the retention times of n-alkanes (C_5_–C_25_) that were run under the same chromatographic conditions. Volatile compound of chromatography results expressed as area percentages were calculated (response factor: 1).

### 3.5. Analysis of Flavonoid and Limonoid by HPLC

The flavonoid and limonoid standards included the following: tangeritin, nobilentin, limonin and nomilin. All the standards were prepared in methanol and stored at −18 °C before use.

The method followed for the quantitative determination of flavonoid standards was previously described by Lin et al. [43]. Aliquots of 20 µL of the filtrate were injected into the injection port and analyzed with an HPLC instrument (Hitachi, Ltd., Tokyo, Japan). The standard and sample were respectively dissolved in methanol to the desired concentrations. The remaining procedures were carried out as previously reported by Lin et al. [43]. HPLC instrument (Hitachi, Japan) was attached to a detector L-2400 UV and a Hitachi L-2130 pump. A column (RP-18GP250 Mightysil (l) 250 mm; i.d., 4.6 mm; thickness, 0.32 µm; Kanto Chemical Co., Inc., Tokyo, Japan) was used for separation. The sample solutions to be analyzed were respectively filtered through a 0.45 µm filter. Aliquots of 20 µL of the filtrate were analyzed. The same mobile phase and elution conditions were adopted. The calibration curves were established for each flavonoid by plotting the peak area vs. each corresponding concentration, from which quantitations of the flavonoid standards were achieved.

The residues were dissolved in 10 mL of acetonitrile and filtered through a 0.45-μm syringe filter. Ten microliters of the filtered solution were then injected into a liquid chromatography column. The remaining procedures were carried out as previously reported [44]. A mixture of methanol, acetonitrile, and water (1:37:62, *v*/*v*/*v*) was used as the mobile phase, and its flow rate was set at 1 mL/min. A UV spectrophotometer detector set at a wavelength of 210 nm was used for detecting limonin and nomilin.

### 3.6. Analysis of Phytosterol Composition by GC

The remaining procedures were carried out as previously reported [38] using an Agilent 6890 GC equipped with a 60 m × 0.25 mm i.d. DB-1 fused-silica capillary column (Agilent, Palo Alto, CA, USA) with a film thickness of 0.25 μm coupled to an Agilent model 5973 N MSD mass spectrometer. The injector temperature was maintained at 250 °C. The carrier gas (helium) flow rate was 1 mL/min. The electron energy was 70 eV at 230 °C. The derivatized phytosterol extracts were analyzed by GC using a 1:30 split ratio injection at 260 °C using hydrogen carrier gas. The initial column temperature was held at 50 °C for 0.5 min and then increased at a rate of 20 °C/min to 320 °C and held for a further 10 min with a flow of 1.4 mL/min. The 5α-cholesterol was taken as an internal standard. Aliquots of 2 μL of this solution were injected into the gas chromatograph, and the ratio of the peak areas of the analyte and internal standard was used as an analytical signal.

### 3.7. Antioxidant Capacity Assay

In order to evaluate the antioxidant activity of the citrus oleoresin, the biochemical methods of DPPH and ABTS radical scavenging assays were used. These tests were carried out in triplicate. The DPPH radical-scavenging activity was measured according to the method of Braca et al. [45]. Samples with different concentrations (5~50 mg/mL) were mixed with methanol solution containing DPPH radicals (0.2 mM). The mixture was shaken vigorously and incubated for 30 min in darkness at room temperature, and then absorbance at 517 nm was measured. The scavenging activity of ABTS radicals (ABTS^+^) was measured using the method of Fellegrini et al. [46], with some modifications. ABTS^+^ were produced by reacting ABTS solution (7 mM) with potassium persulphate (1.4 mM), and the mixture was kept in the dark at room temperature for 16 h. At the time of use, the ABTS^+^ solution was diluted with methanol (475 mL) to an absorbance of 0.75 ± 0.02 at 734 nm. Samples with different concentrations (1~25 mg/mL) were then added to the ABTS^+^ solution and mixed vigorously. After reaction at room temperature for 5 min, the absorbance at 734 nm was measured.

The oxidation stability of citrus oleoresin in sunflower oil (1 mg/mL) was previously determined as the induction period (IP, hours) that was recorded using a Rancimat 743 (Herisau, Swizerland) apparatus and 5 ± 0.05 g sample of oil at 110 °C with an air flow of 10 L/h [39]. The oil samples that were used to determine the oxidative stability were also analyzed for their volatile oxidation compounds. The oil stability index (OSI) of the oil samples were automatically recorded and taken as the break point of the plotted curves (the intersection point of the two extrapolated parts of the curve). The IP was evaluated on oils in the presence and absence (test) of citrus oleoresin extracted via SFE (1 mg/mL of oil). The antioxidative index (AI) was calculated using the equation AI = IPs/IPo, where IPs is the induction period of oil with citrus oleoresin addition and IPo is the induction period of oil alone.

### 3.8. Statistical Analysis

All experiments were performed in triplicate and all data were expressed as a mean ± standard deviation of the mean (SD). Data obtained in the same group were analyzed by analysis of variance (ANOVA) with the computer statistical software SPSS 10.0 (SPSS, Chicago, IL, USA). Duncan’s multiple range tests were used to test the significance of the differences between paired means. The significance of each difference was judged by a confidence level of *p* < 0.05 code.

## 4. Conclusions

Important variations in the volatile compounds found in the oleoresin samples extracted from the three citrus varieties were identified. The results could, based on the indicated essential oil compositions, explain the perceived differences in the characteristic aroma of each of the fruits and provide useful chemical quality information applicable for the improvement and utilization of various varieties of citrus fruits. SFE with ethanol was used to prepare the oleoresin samples extracted from the three citrus varieties because it would not cause thermal degradation and because the residues of the organic solvents contained a diversity of compounds. In terms of volatile compounds, Murcott oleoresin with higher levels of oxygenated compounds indicated a better sensory quality. In term of non-volatile compounds, Ponkan oleoresin with higher levels of PMF exhibited considerable antioxidant capacity, which was helpful on the physiological synergistic effect of limonoids and phytosterols. The activities of the compounds found in oleoresin have already been demonstrated by previous studies to have important biological applications, such that these compounds are products of interest to both the food and pharmaceutical industries.

## Figures and Tables

**Table 1:** Volatile constituents (%) of citrus oleoresin.

Compounds	RI ^a^	Tankan ^b^	Ponkan ^b^	Murcott ^b^
**Monoterpenes**				
α-thujene	933	N.D ^c^	0.74	0.43
α-pinene	937	2.13	2.60	2.39
sabinene	968	0.60	1.12	0.93
β-pinene	979	0.07	1.21	0.72
β-myrcene	980	5.81	4.86	5.68
1-phellandrene	1002	0.21	0.23	0.20
p-cymene	1004	N.D	1.69	0.84
limonene	1037	86.13	72.72	76.34
β-ocimene	1052	0.43	0.24	0.20
γ-terpinene	1063	0.17	8.77	6.01
α-terpinolene	1085	0.10	0.50	0.10
**Sesquiterpenes**				
α-copaene	1353	0.06	N.D	0.12
γ-elemene	1380	0.21	N.D	N.D
γ-cadinene	1389	0.11	N.D	N.D
β-caryophyllene	1428	0.37	0.16	0.49
γ-muurolene	1481	0.08	N.D	0.14
germacrene D	1478	0.09	0.26	0.18
δ-cadinene	1497	0.18	N.D	0.15
**Aldehydes**				
nonanal	900	0.06	1.52	0.79
decanal	1000	0.07	0.78	1.14
2-decenal	1021	0.07	N.D	N.D
perillaldehyde	1042	0.05	0.10	0.12
undecanal	1100	N.D	0.21	0.17
citronellal	1132	N.D	0.36	0.50
neral	1210	N.D	0.21	0.31
β-sinensal	1673	0.18	0.20	N.D
α-sinensal	1689	0.38	N.D	0.18
**Alcohols**				
linalool	1098	1.40	0.81	1.05
α-terpineol	1177	0.44	0.11	0.22
**Esters**				
geranyl acetate	1362	0.16	N.D	N.D
citronellyl acetate	1382	0.21	N.D	0.20
neryl acetate	1434	0.18	N.D	0.20
**Ketone**				
carvone	1217	N.D	0.32	0.18
^d^ **Total terpene compound**		**96.75**	**95.11**	**94.92**
^e^ **Total oxygenated compound**		**3.25**	**4.89**	**5.08**

^a^ RI: Retention index; ^b^ identified via comparison of the mass spectra with the RI; ^c^ N.D: not detected; ^d^ Total terpene compound contains Monoterpenes and Sesquiterpenes; ^e^ Total oxygenated compound contains Aldehydes, Alcohols, Esters and Ketone.

**Table 2:** Non-volatile composition of citrus oleoresin.

Compounds	Tankan	Ponkan	Murcott
**Polymethoxyflavones (mg/g)**			
nobilentin	62.23 ± 4.62 ^a^	195.45 ± 5.42 ^c^	99.31 ± 3.44 ^b^
tangeritin	24.05 ± 1.44 ^a^	64.12 ± 3.11 ^c^	54.19 ± 2.17 ^b^
**Limonoids (mg/g)**			
limonin	71.17 ± 2.45 ^a^	316.02 ± 17.39 ^c^	117.83 ± 6.34 ^b^
nomilin	41.53 ± 1.62 ^a^	90.24 ± 5.32 ^b^	130.47 ± 4.25 ^c^
**Phytosterols (μg/g)**			
campesterol	211.62 ± 8.94 ^a^	304.77 ± 24.38 ^b^	455.54 ± 25.83 ^c^
stigmasterol	131.85 ± 5.52 ^a^	680.67 ± 42.15 ^c^	374.17 ± 31.59 ^b^
lanosterol	189.34 ± 7.63 ^a^	N.D	486.71 ± 32.54 ^b^
fucosterol	153.33 ± 9.28 ^a^	N.D	N.D

^a^ Data presented are in mean ± SD (*n* = 3), with the different letters indicating significant differences (*p* < 0.05).
